# Precise Motion Control of a Power Line Inspection Robot Using Hybrid Time Delay and State Feedback Control

**DOI:** 10.3389/frobt.2022.746991

**Published:** 2022-02-24

**Authors:** Ahmad Bala Alhassan, Xiaodong Zhang, Haiming Shen, Haibo Xu, Khaled Hamza, Gilbert Masengo

**Affiliations:** ^1^ School of Mechanical Engineering, Xi’an Jiaotong University, Xi’an, China; ^2^ Shaanxi Province Key Laboratory of Intelligent Robot of Xi’an Jiaotong University, Xi’an, China

**Keywords:** power line, robotic inspection, motion control, oscillation control, time delay (TD)

## Abstract

Intelligent robotic inspection of power transmission lines has proved to be an excellent alternative to the traditional manual inspection methods, which are often tedious, expensive, and dangerous. However, to achieve effective automation of the robots under different working environments, the dynamic analysis and control of the robots need to be investigated for an efficient inspection process. Nonetheless, the application of control techniques for the position, speed and vibration control of these robots has not been explored in detail by the existing literature. Thus, an approach for precise motion control of the sliding inspection robot is presented in this paper. The main contribution of the study is that the chattering problem associated with the traditional command shaping time delay control (TDC) was minimized by smoothing the chattered input signal. Then, the improved control (iTDC) which is effective for oscillation control is hybridized with a pole placement based feedback control (PPC) to achieve both position and the sway angle control of the robot. The nonlinear and the linearized models of the sliding robot were established for the control design and analysis. Three parameters of the robot, namely, the length of the suspended arm, the mass of the payload, and the friction coefficient of different surfaces, were used to assess the robustness of the controller to model uncertainties. The iTDC + PPC has improved the velocity of TDC by 201% and minimizes the angular oscillation of PPC by 209%. Thus, the results demonstrate that the hybridized iTDC + PPC approach could be effectively applied for precise motion control of the sliding inspection robot.

## Introduction

As the world is continuously becoming over-dependent on electricity, any power transmission failure could cause a catastrophic impact on people’s livelihood, including national security, health system, education, and economy. Thus, to ensure stable and reliable electricity transmission from the generation stations to consumers, the power line cables and its supporting equipment need to be routinely monitored for early fault detection and maintenance (Chen and Wang, 2021; Chen et al., 2021). Effective identification and localization of faults on the power line system are crucial as they significantly minimize the maintenance cost and avoid unnecessary power outages. Traditionally, most of the power line inspection tasks were carried out manually by linemen (line crawling), ground cranes, and telescopes, which are often dangerous, ineffective, slow, and expensive (Jalil et al., 2019; Gao et al., 2020).

Interestingly, with the introduction and advancement of intelligent robotics, the power transmission line inspection robots gained a lot of recognition and have been in constant development to replace the tedious manual inspection approaches (Menendez et al., 2017). These inspection robots can be classified into three, namely, the ground robots for the inspection of substations (Wang et al., 2012, Wang et al., 2019, Wang et al., 2020), the flying robots (UAVs) (Máthé and Buşoniu, 2015; Shakhatreh et al., 2019), and the suspended robot that climbs and slide along the power line. Although the flying robots (UAVs) were also deployed to inspect the power lines, the existing research focused on the climbing robots due to their proximity to the lines. Hence, they provide more accurate inspection results. Thus, among the most advanced power line inspection (PLI) robots that have been deployed for live power line inspection includes the Linescout of Hydro Quebec research institute, Canada (Pouliot and Montambault, 2011; Pouliot et al., 2015), Expliner of HiBot Corporation, Japan (Debenest and Guarnieri, 2010), TI of American Electric Power Research Institute (Phillips et al., 2012), POLIBOT (Limall et al., 2018), LineRanger (Richard et al., 2019), LineBot (Wang et al., 2013), LineDrone (Hamelin et al., 2019), and LineRover (Zhao et al., 2010). In addition, the types and developmental trends of the intelligent robotic power line inspections were reviewed in (Zhang and Dai, 2021). However, these robots were often heavy and difficult to be placed on the power line or had complex operating mechanisms. Thus, the robot presented in this work is lightweight and can be automated easily.

Furthermore, due to the complexity of the working environment and the impact of external disturbances, a control algorithm is highly required to stabilize the automation of the robot along the power line. Nonetheless, most of the current inspection robots only set up the control system for the robot’s communication on the line and the ground control unit (GCU) as a teleoperated control. Debenest et al. (2008), presented an example of the teleoperated control for the Expliner robot, whereby two omnidirectional antennas were placed on the robot body and one-directional antenna on the GCU. The robot motion can be controlled by the directional antennal, joysticks, and manual switches on the GCU. Yet, this type of control can only serve for the communication of the robot and its operator, motion planning, or a start and stop operation of the robot.

Thus, it is essential to comprehensively study the dynamic behavior and control of the PLI robots for effective automation of the robots along the power line. Nonetheless, some studies presented the control and analysis of the inspection robot for a specific operation of the robot. For instance, Shruthi et al. (2019a); Shruthi et al. (2019b) presented an optimal crossing control of a dual-arm biotic inspection robot. The study performs the kinetic and dynamic analysis of the robot for crossing tension towers through jumper wires. Another dynamic and kinematic analysis for a specialized cable-climbing robot was presented for the inspection of cable-stayed bridges (Xu et al., 2015). The GA-based PID control was designed and simulated for the path crossing control. A PID-based position control of an inspection robot was designed and implemented using a robot operating system (ROS) platform (Xu et al., 2019). As one of the vital aspects of the power line inspection, some studies analyzed the obstacle avoidance process using fuzzy logic control (Jin, 2013; Li and Choi, 2013).

Tao Zhao and Dian investigate the stabilization problem of nonlinear systems under model parameter variations and time-varying delays using fuzzy logic control (Zhao and Dian, 2018). Also, the authors established the simplified dynamic model of a 2DoF PLI robot for the balance adjustment posture. They analyzed its performance using the adaptive gain-scheduled backstepping control (Dian et al., 2019) and the Fuzzy Gain Scheduling PID Controller (Zhao et al., 2020). Wei et al. (2021) presented the stability analysis of a dual-arm inspection robot moving along a catenary power line. The study established that for a stable inspection of a flexible power line, the walking posture of the robot should be changeable in relation to the slope of the line. Moharam et al. (Korayem et al., 2010) investigate the payload carrying capacity of the inspection robot using a feedback linearization control approach on both flexible and rigid power cables. In (Kakou et al., 2021; Kakou and Barry, 2022), a PID-based control of a dual-purpose robot for vibration suppression and inspection of power lines was presented. Yang et al. (2021) presented an optimal control technique for preventing the overheating of the inspection robot’s walking motor under unpowered downslope speed. As most of the mathematical models only partially represented the dynamics of the physical systems, the performance of the model-based controllers could be effected during practical applications or due to uncertain disturbances. In (Tutsoy and Barkana, 2021), a model-free adaptive control scheme was introduced to handle model and environmental uncertainties. Also, large control actions and digital chattering could cause chaotic dynamics in the control systems. Thus, a reinforcement learning based approach was utilized to investigate the chaotic dynamics associated with digital chattering (Tutsoy and Brown, 2016).

However, those studies did not provide comprehensive dynamic modelling and control of the complete PTLI robot for sliding inspection process. Thus, to develop a more precise motion control of the robot, its position, speed, and angular displacement need to be controlled efficiently. As reported in our recent in-depth review of the robotic power line inspection (Alhassan et al., 2020), it was highlighted that the current inspection robots had not studied the dynamic analysis and precise motion control of the robot on the power line in detail. In this paper, the command shaping time delay control (TDC) and the pole placement based state feedback control (PPC) were designed and investigated for the precise motion control of the dual-arm PLI robot. The TDC has been proven to suppress the oscillation of the flexible systems effectively. The TDC is a typical pre-filter whereby a series of impulses is convoluted with the input command to generate shaped input, effectively eliminating the system vibration or oscillation mode (Ha et al., 2018).

The main advantage of the TDC is that it does not need continuous feedback and only requires the natural frequency and damping ratio of the system, which can either be estimated using the system output response or from the system model. TDC is usually used to control continuous-time systems from unnecessary oscillations such as crane control, vibration control, and robot control (Mohammed et al., 2020). On the other hand, PPC is the process of changing the location of the poles of the uncontrolled system to a stable region. The main advantage of the PPC is that the desired performance of the control system, namely, overshoot and settling time, can be pre-defined based on the system behavior. Though it is a model based controller, PPC has been applied effectively for numerous control systems, including motor position control (Ahmad et al., 2018; Iswanto and Ma’arif, 2020), process control (Ramezani Khosro and Fatehi, 2020), virtual synchronous generators control (Pourmohammad et al., 2020), and other multivariable systems (Abdelaziz, 2017; Mammadov, 2021).

Thus, this work presents a precise motion control of an inspection robot on power transmission line, which utilizes the advantages of the TDC and the PPC. However, the traditional TDC has chattering problem due to the number of delays added to the control signal. Thus, the main innovation of this paper of this paper is that the traditional TDC was improved (iTDC) by smoothing the input signal with first order filter which minimized the chattering problem of the TDC. Also, the iTDC was hybridized with a PPC to achieve a precise position and oscillation control of the dual-arm PTLI robot. The dynamic model of the sliding robot was initially established, and then the controllers were designed based on the model. The TDC was designed using the natural oscillatory response of the uncontrolled system for the oscillation suppression, while the PPC was designed for the position control. Simulation analysis was conducted to assess the effectiveness of the designed controllers. Moreover, a robustness analysis was carried out to investigate the influence of model uncertainties on the robot.

The paper is divided into five sections. The second section presents the description of the system and its dynamics. The third section presents the control design while the simulation analysis and discussions were presented in section four. Finally, the summary of the complete study is given in section five.

## System Description and Dynamic Modeling

### The Dual-Arm PLI Robot

**FIGURE 1:**
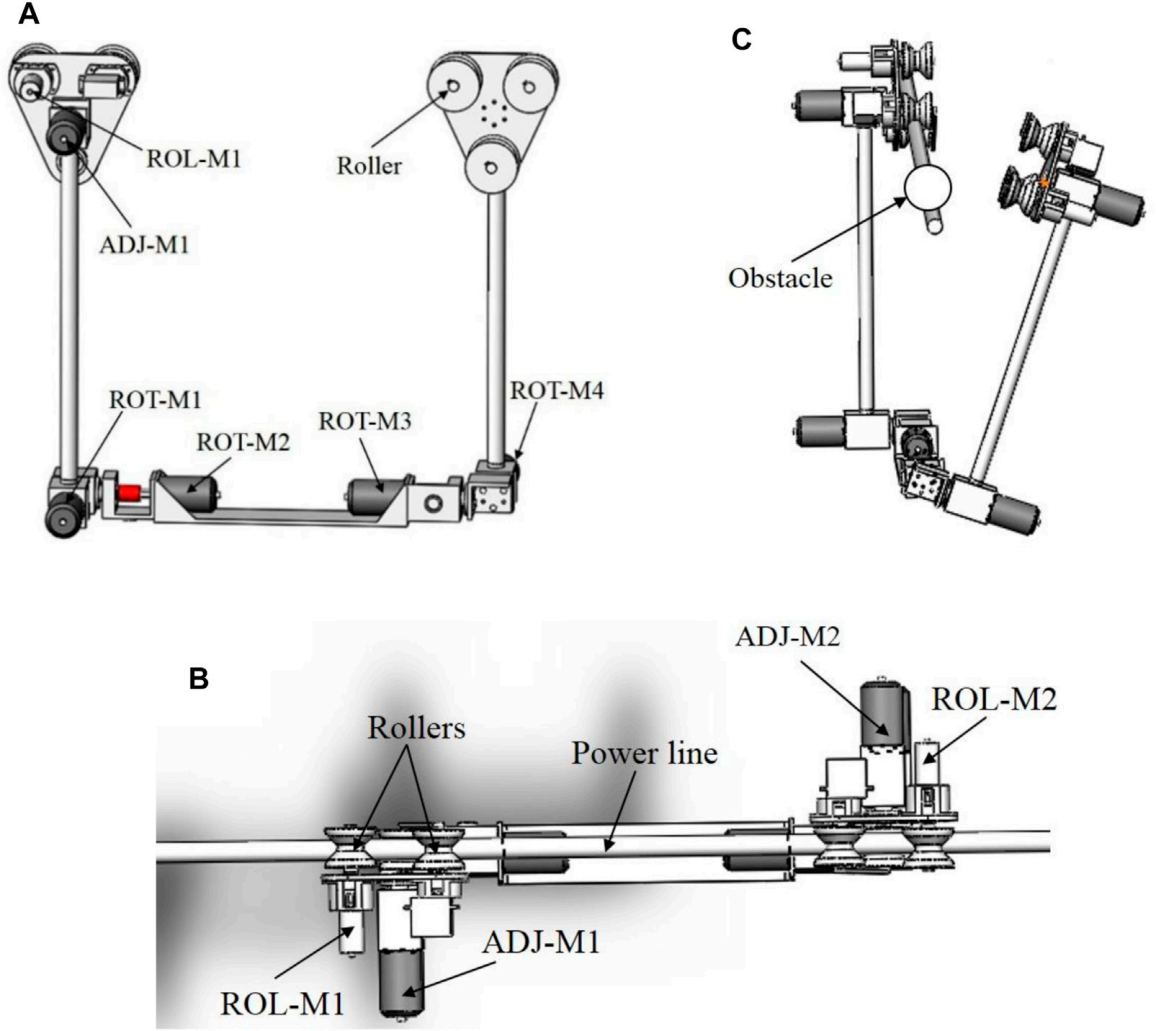
The structure of the dual-arm PTLI robot. **(A)** Side view; **(B)** Obstacle avoidance; **(C)** Plan view. The system has eight motors, two motors (ROL-M1 and2) drives the wheels, two motors (ADJ-M1 and 2) aligns the grippers on the line, and four motors (ROT-M1–4) adjusts the arms.

The PLI robot considered in this work is illustrated in the conceptual design of Figure 1A. As shown, the robot is a dual-arm robot that climbs and rolls along the power transmission line for the inspection process. The structure is chosen due to its proximity to the line that provides improved stability and high inspection accuracy compared to the flying PLI robots. The system has two identical arms, two triangular grippers, and a rectangular trunk. Therefore, the automation of the robot is operated by eight motors. The gripper system comprises two motors (ROL-M1 and ROL-M2) that drive the wheels along the line and another two motors (ADJ-M1 and ADJ-M2) that adjust the grippers for appropriate positioning the wheels on the line. The remaining four motors (ROT-M1–4) adjusts the cylindrical arms, especially during the obstacle avoidance process. The arrangement of the wheels and other parts of the robot is illustrated by the plan view of the robot-line system of Figure 1B. Moreover, the trunk or the robot’s base housed the electronic circuits, the onboard battery, and other payloads. Thus, to achieve smooth motion of the robot along the line, three wheels are attached to each gripper that carries the whole robot body. Amongst the three wheels of each arm, two wheels, including the driven wheel, climb the power line while the other wheel provides support when moving along an inclined cable.

In addition, as the power line system is associated with many obstacles, the robot should be able bypass them. The robot can either roll over the obstacles or bypass them by lifting its arms. Figure 1C demonstrated the objectives of the adjustment motors for an obstacle bypassing process (circular warning ball), whereby the front arm is raised and rotated away from the power line. As illustrated, once the robot is near the obstacle, the robot stops, and the front adjustment motors (ROT-M1 and 2) raise the front arm from the line, and then the ADJ-M1 rotates the arm away from the line. The rear arm then moves the robot towards the obstacle to allow the front arm to avoid the obstacle. Then, the front arm will be re-climbed to the line using ADJ-M1 and ROTM1 and 2. At this moment, the obstacle is in the middle of the arms. Therefore the same process is repeated for the rear-arm using ROT-M3 and 4 and ADJ-M2 to bypass the obstacle finally. However, the scope of this work focuses on the motion control of the robot on the power line for the sliding inspection process.

### Dynamic Modelling

**FIGURE 2:**
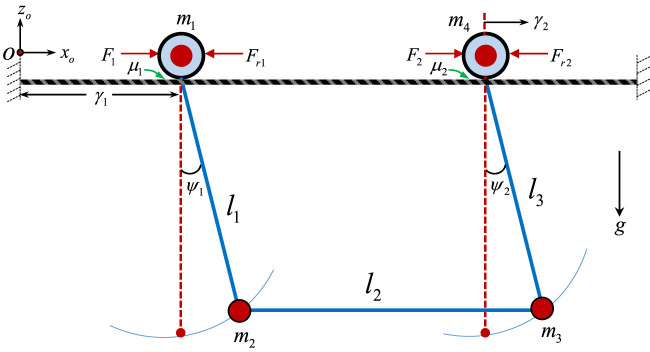
The schematics of the dual-arm PTLI robot represented as two rollers attached to two coupled cylindrical arms.

To design and assess any control algorithm, a dynamic model of the system is highly required. Here, the dual-arm robot is represented by considering the two cylindrical arms to exhibit oscillatory motion when the robot moves along the transmission line, as illustrated in Figure 2. As shown, the forces (F_1_ and F_2_) that move the two wheels along the line from a reference point, O, cause the arms to oscillate for small angular displacements (ψ_1_ and ψ_2_) for arm 1 and 2, respectively. Also, the two symmetrical arms of lengths *l*_1_ and *l*_3_ were coupled by a horizontal rectangular base of length *l*_3_. The masses m_1_ and m_4_ of the wheels move to a horizontal distance of γ_1_ and γ _2_, respectively. The masses of the arms (m_2_ and m_3_) were considered to be concentrated at the base of the robot.

Moreover, as the robot moves along the power line, which is not smooth, the effect of frictional force cannot be neglected. This frictional force which is the resistive force between the two wheels and the power line prevents the wheels from moving freely along the lines. Thus, it is very important to incorporate the effects of friction into the derived model for a more realistic analysis (Li et al., 2017).

**FIGURE 3:**
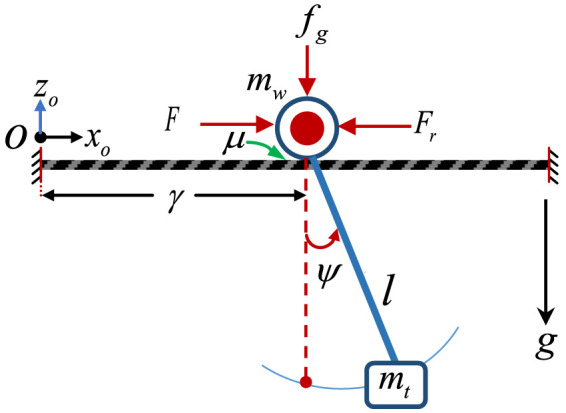
The simplified schematics of the robot represented as a single roller carrying a payload.

However, since the robot is symmetrical and perfectly coupled at the base, the angular displacement of the first arm and the second arm can be considered to be the same. Thus, the schematics of the robot shown in Figure 3 can sufficiently describe the behavior of the system. Therefore, the force (*F*) that moves the wheel along the line from a reference point, *O* to a distance, γ, causes the arm to oscillate for small angular displacement, ψ. Here, the two wheels move with the same speed and experienced similar angular displacement. Therefore, the mass of the wheel, *m*_*w*_, is the total combined masses of the two wheels, while the mass of the arms, *m*_*t,*_ was considered to be concentrated at the base of the robot.

Moreover, the Lagrangian formulation of Eq. 1 was utilized to derive the robot’s dynamic model, where *m*_*i*_ is the mass, *Q*_*i*_ is the total non-conservative forces, and *q*_*i*_ is the independent point for *i*th coordinates. Then the total kinetic (T) and potential (U) energies given in Eq. 2 can be expressed in Eq. 3 and Eq. 4, respectively, for the robot.

$$\frac{d}{dt}\left(\frac{dT}{d{\dot{q}}_{i}}\right)-\frac{dT}{dq_{i}}+\frac{dU}{dq_{i}}=Q_{i}\;;\;i=1,2,3\ldots n$$ (1)

$$T=\sum_{i=1}^{n}\frac{1}{2}m_{i}{\dot{q}}_{i}^{2}\;;\;U=\sum_{i=1}^{n}\left(m_{i}gh_{i}\right)$$ (2)

$$T=\frac{1}{2}m_{w}{\dot{\gamma }}^{2}+\frac{1}{2}m_{t}\left({\dot{\gamma }}^{2}+{\dot{\psi }}^{2}l^{2}\cos^{2}\psi +2\dot{\gamma }\dot{\psi }l\cos\psi \right)$$ (3)

$$U=-m_{t}gl\cos\psi$$ (4)

By solving Eq. 1 using Eq. 3 and Eq. 4, the generalized equation for each coordinate are expressed for the whole dynamic of the robot as shown in Eq. 5 and Eq. 6:

$$\left(m_{w}+m_{t}\right)\ddot{\gamma }+m_{t}l\ddot{\psi }\cos\psi -m_{t}l{\dot{\psi }}^{2}\sin\psi =F-F_{r}$$ (5)

$$m_{t}l\ddot{\gamma }\cos\psi +m_{t}l^{2}\ddot{\psi }\cos^{2}\psi +m_{t}gl\sin\psi =0$$ (6)

Also, to represent the dynamics in state space for the control design, the nonlinear models should linearized about a small angular displacement. Thus, using the Taylor’s series approximation, the angle can be linearized using Eq. 7.

$$\sin\psi_{i}≃\psi_{i}\;;\;\cos\psi_{i}≃1\;;\;i=1,2$$ (7)

Moreover, as illustrated in schematics, the linear force that derives the robot along the power line is produced by the DC motor connected to the wheels. Thus, to incorporate the motor dynamics into the derived model, the relationship between the motor torque, Tm, reference voltage, V_ref,_ and the linear force, F shown in Eq. 8 and Eq. 9 are utilized, where r_p_ is the radius of the wheel or pulley, R_m_ is the motor electrical resistance, T_m_ is the DC motor torque, k_m_ is the motor torque constant, k_e_ is the motor electrical constant, and ω_m_ is the motor angular velocity as described (Shehu et al., 2019).

$$T_{m}=r_{p}F=\frac{k_{m}}{R_{m}}V_{ref}-\frac{k_{m}k_{e}}{R_{m}}\omega_{m}$$ (8)

$$\omega_{m}=\frac{\dot{\gamma }}{r_{p}}$$ (9)

Finally, the state space representation of the system after substituting the motor dynamics and the friction can be expressed based on the controllable form of Eq. 10 in Eq. 11, where y_opt_ is the output matrix containing the robot’s position, velocity, and angular displacement. The constant parameters *A*, *B*, and *C*, are the system matrix, input matrix, and output matrix, respectively, for the states variables, *z*, input vector, *u*. The parameters of the model are illustrated in Table 1.

$$\dot{z}\left(t\right)=Az+Bu\left(t\right)\;;\;y_{op}=Cz\left(t\right)$$ (10)

$$\left[\begin{array}{c} \dot{\gamma }\\ \ddot{\gamma }\\ \dot{\psi }\\ \ddot{\psi } \end{array}\right]=\left[\begin{array}{cccc} 0 & 1 & 0 & 0\\ 0 & -\left(\frac{f_{v}}{m_{w}}+\frac{k_{m}k_{e}}{m_{w}R_{m}r_{p}^{2}}\right) & \frac{m_{t}g}{m_{w}} & 0\\ 0 & 0 & 0 & 1\\ 0 & \left(\frac{f_{v}}{m_{w}l}+\frac{k_{m}k_{e}}{m_{w}R_{m}r_{p}^{2}l}\right) & \frac{-\left(m_{w}+m_{t}\right)g}{m_{w}l} & 0 \end{array}\right]\left[\begin{array}{c} \gamma \\ \dot{\gamma }\\ \psi \\ \dot{\psi } \end{array}\right]+\left[\begin{array}{c} 0\\ \frac{k_{m}}{m_{w}R_{m}r_{p}}\\ 0\\ -\frac{k_{m}}{m_{w}R_{m}r_{p}l} \end{array}\right]\left(V_{ref}\right)\;y_{opt}=\left[\begin{array}{cccc} 1 & 0 & 0 & 0\\ 0 & 1 & 0 & 0\\ 0 & 0 & 1 & 0 \end{array}\right]\left[\begin{array}{c} \gamma \\ \dot{\gamma }\\ \psi \\ \dot{\psi } \end{array}\right]$$ (11)

**TABLE 1:** Model parameters of the robot.

Parameter	Value	Unit	Description
*l*	0.45	m	Length of cylindrical arm
m_w_	2.40	kg	Mass of the wheel
m_t_	3.80	kg	Combined mass of arm and trunk
r_p_	0.03	m	Radius of the wheel or pulley
γ	—	m	Robot linear position
ψ	—	deg	Robot angular displacement
R_m_	0.46	Ω	Motor electrical resistance
k_m_	0.18	Nm/A	Motor torque constant
k_e_	0.29	Vs/rad	Motor electrical constant

## Control Approaches

This section presents the comprehensive design and analysis of the control algorithms for the dual-arm robot’s oscillation, position, and speed control. The analysis includes time delay control to control the angular displacements (oscillations) and the pole placement-based feedback control for the precise position and speed control. However, before designing these kinds of controllers, it is required to check whether the system under consideration can be controlled. The controllability of the linearized system can be calculated using the controllability matrix, G_c_, given in Eq. 12. After testing with the parameters of Eq. 11, the determinant of the matrix is non-zero, which confirms that the system is controllable, and thus, the controllers can be designed.

$$G_{c}=\left[\begin{array}{cccc} B & AB & A^{2}B & A^{3}B \end{array}\right]\;;\;\left|G_{c}\right|\neq 0$$ (12)

### Time Delay Based Oscillation Control Approach

**FIGURE 4:**
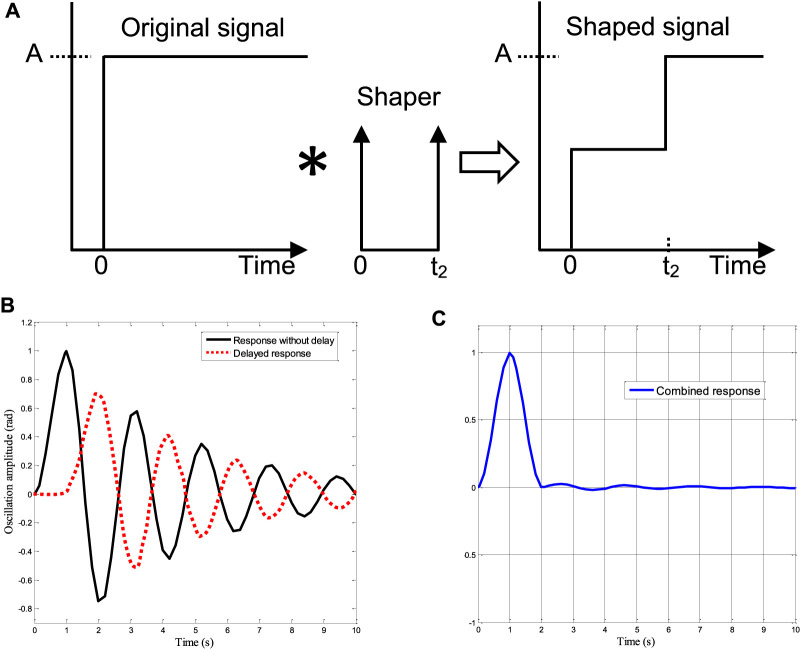
The process of time delay control: **(A)** Block diagram; **(B)** Convolution of two responses; **(C)** Resulting cancelled oscillation.

The time delay method reduces the oscillation of the payload by delaying some part of the command signal before feeding to the system. The delayed signal cancelled out the effect of the un-delayed signal leading to a zero oscillation (ZO). TDC is one of the cheapest control methods that provide oscillation control of flexible systems without redesigning the physical system. TDC is simply designed using the estimated damping ratio and natural frequency of the system. The process of TDC containing two impulses (ZO) is demonstrated in Figure 4.

To obtain the appropriate amplitude and their respective time delays of the TDC, the general representation of the oscillation amplitude of a second-order underdamped system of Eq. 13 or Eq. 14 in the time domain is considered, where *A*_*i*_ is the *i*th impulse amplitude, *t*_*i*_ is the *i*th impulse, ω is the natural frequency, and ζ is the damping ratio of the system as presented in our TDC analysis on rotary cranes (Alhassan et al., 2018).

$$G\left(s\right)=\frac{\omega^{2}}{s^{2}+2\zeta \omega s+\omega^{2}}$$ (13)

$$A=\frac{\omega }{\sqrt{\left(1-\zeta^{2}\right)}}e^{-\zeta \omega t_{i}}\sqrt{{\left(\sum_{i=1}^{n}A_{i}e^{\zeta \omega t_{i}}\cos\left(\omega t_{i}\sqrt{\left(1-\zeta^{2}\right)}\right)\right)}^{2}+{\left(\sum_{i=1}^{n}A_{i}e^{\zeta \omega t_{i}}\sin\left(\omega t_{i}\sqrt{\left(1-\zeta^{2}\right)}\right)\right)}^{2}}$$ (14)

The non-dimensional amplitude of oscillation can be determined by dividing Eq. 14 by the oscillation amplitude of a single impulse of unity magnitude. Thus, the amplitude of the residual oscillation from the applied single unity magnitude at rest is given in Eq. 15. Thus, dividing Eq. 14 and Eq. 15 gives the residual vibration as in Eq. 16. This shows the amount of oscillations generated by an impulse sequence for any given frequency of an underdamped system (ζ< 0). Depending on the chosen constraints, Eq. 16 can be equated to calculate the amount of tolerable oscillation. Assuming no residual oscillation is needed (i.e. zero oscillation after the last impulse), then R_1_ and R_2_ of Eq. 17 should be directly set to zero. This is called zero oscillation (ZO) constraint. To obtain a similar rigid body motion of the original command signal, the summation of the TDC’s amplitudes of total impulses should be unity, as shown in the summation constraint of Eq. 18.

$$A_{↑}=\frac{\omega }{\sqrt{\left(1-\zeta^{2}\right)}}$$ (15)

$$V\left(\omega ,\zeta \right)=\frac{A}{A_{↑}}=e^{\zeta \omega t_{i}}\sqrt{{\left(R_{1}\right)}^{2}+{\left(R_{2}\right)}^{2}}$$ (16)

$$R_{1}=\sum_{i=1}^{n}A_{i}e^{\zeta \omega t_{i}}\cos\left(\omega t_{i}\sqrt{\left(1-\zeta^{2}\right)}\right)\;;\;R_{2}=\sum_{i=1}^{n}A_{i}e^{\zeta \omega t_{i}}\sin\left(\omega t_{i}\sqrt{\left(1-\zeta^{2}\right)}\right)$$ (17)

$$\sum_{i=1}^{n}A_{i}=1$$ (18)

In addition, to avoid unwanted response delay, the time of application of the first impulse is set at *t*_1_ = 0. Therefore, to design a ZO, two impulse sequences are needed. However, the ZO TDC does not justify the robustness to parameter errors. This robustness can be improved by equating the derivatives of both *R*_*1*_ and *R*_*2*_ to zero, which will produce small changes in oscillation in relation to the parameter errors. In general, the derivative (D) of the residual oscillation has the form of Eq. 19. The TDC can also take the form of ZO(D)^i^, with i≥0  as the derivate order. To design ZODD TDC, the second derivative of R_1_ and R_2_ is considered, i.e., i=2 . Thus, solving the constraints gives the four impulse ZODD TDC’s parameters as in Eq. 20:

$$\frac{\partial^{i}R_{1}}{\partial \omega^{i}}=0\;;\;\frac{\partial^{i}R_{2}}{\partial \omega^{i}}=0$$ (19)

$$\begin{array}{l} \left[\begin{array}{c} A_{i}\\ t_{i} \end{array}\right]=\left[\begin{array}{cccc} \frac{1}{{\left(1+k\right)}^{3}} & \frac{3k}{{\left(1+k\right)}^{3}} & \frac{3k^{2}}{{\left(1+k\right)}^{3}} & \frac{k^{3}}{{\left(1+k\right)}^{3}}\\ 0 & \tau_{d} & 2\tau_{d} & 3\tau_{d} \end{array}\right]\\ \tau_{d}=\frac{\pi }{\omega \sqrt{\left(1-\zeta^{2}\right)}}\;;\;k=e^{\frac{-\pi \zeta }{\sqrt{\left(1-\zeta^{2}\right)}}} \end{array}$$ (20)

**FIGURE 5:**
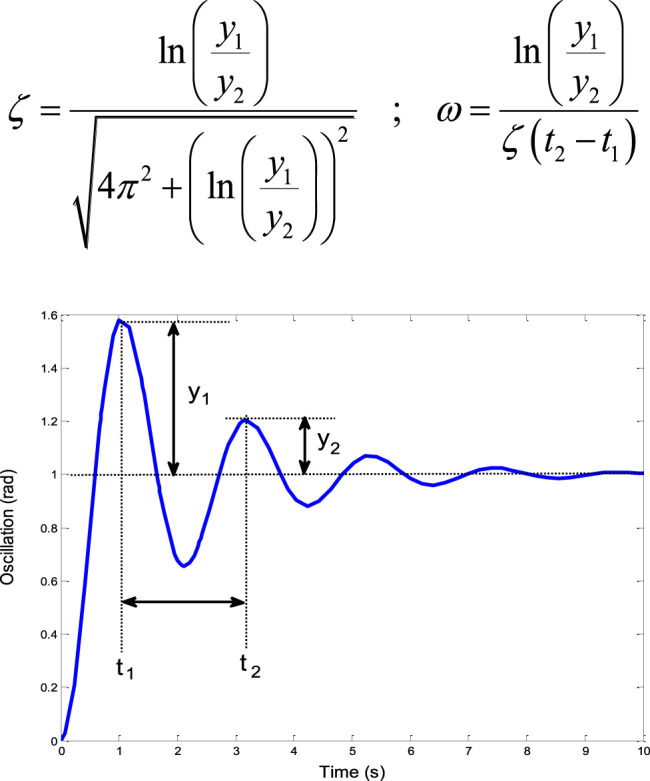
The process of calculating the natural frequency and damping ratio from a response curve.

Thus, the parameters of Eq. 20 are the time delay control (TDC). Moreover, the most important parameters for the design of any TDC are the natural frequency and damping ratio of the uncontrolled system. In this study, a logarithmic decrement approach is employed due to its effectiveness and simplicity in determining the natural frequency and damping ratio directly from the time response curve. To estimate the damping ratio of the system of Figure 5, any two successive peaks can be selected, and the corresponding control parameters can be calculated using Eq. 21 (Ha and Kang, 2013).

$$\zeta =\frac{\ln\left(\frac{y_{1}}{y_{2}}\right)}{\sqrt{4\pi^{2}+{\left(\ln\left(\frac{y_{1}}{y_{2}}\right)\right)}^{2}}}\;;\;\omega =\frac{\ln\left(\frac{y_{1}}{y_{2}}\right)}{\zeta \left(t_{2}-t_{1}\right)}$$ (21)

### Pole Placement Based Position Control Approach

Inherently, an uncontrolled system is unstable or at least marginally stable, with its poles located either on the right half s-plane or the imaginary axis. These kinds of systems can be controlled by adjusting their poles to the left half-plane (stable region), which is the main objective of any control algorithm. Interestingly, pole placement control (PPC) is one of the control strategies for adjusting the poles to the stable region. The main advantage of the PPC is that the designer can pre-determine the desired poles locations of the closed-loop system in the s-plane based on the required performance criteria.

The percentage overshoot (POS) and the settling time (T_s_) given in Eq. 22 and Eq. 23 are the two essential design requirements for the PPC algorithms, where ξ_c_ and ω_c_ are the damping ratio and natural frequency of the desired closed-loop system, respectively. For our system, it is required that the robot smoothly moves along the power line to the desired reference position. Since a precise positioning is needed that allows the robot to stops at the desired position, the POS and the T_s_ should be set to achieve the desired goal:

$$POS=e^{\frac{-\pi \zeta_{c}}{\sqrt{1-\zeta_{c}^{2}}}}\cdot 100\%$$ (22)

$$T_{s}=\frac{4}{\zeta_{c}\omega_{c}}$$ (23)

Thus, the calculated parameters can be substituted in Eq. 24 to generate the closed-loop poles of the new system. However, with this result, only the poles of a second-order system can be obtained. To get the remaining two poles to make the fourth-order system, it is recommended that the calculated poles be made the dominant poles while the other two be located at least 10 times farther away from calculated poles:

$$\lambda_{1,2}=-\zeta_{c}\omega_{c}\pm j\left(\sqrt{1-\zeta_{c}^{2}}\right)$$ (24)

### New Approach Based on Hybrid TDC + PPC

In this section, the hybridized TDC + PPC approach is discussed. In a stand-alone configuration, the TDC has been proven to suppress the oscillation of the flexible systems effectively. The TDC is a typical pre-filter whereby a series of impulses is convoluted with the input command to generate shaped input, effectively eliminating the system vibration or oscillation mode (Ha et al., 2018). To find the control parameters of the TDC for the robot oscillation control, the dynamics of the system is driven by a step input and the corresponding responses are recorded. The formulation of Eq. 21 was then used to generate the natural frequency (4.6002 Hz) and damping ratio (0.0143) of the system. These parameters were then substituted in the TDC formulation of Eq. 20 to generate the desired control gains. Although, the TDC provides angular sway control, it has poor position control and the delayed signal which is fed to the system lead to chattering. Thus, TDC was improved (iTDC) by added a first order filter to the feed-forward path to smooth the input signal, as shown in Eq. 25.

$$\left[\begin{array}{c} A_{i}\\ t_{i} \end{array}\right]=\left[\begin{array}{cccc} 0.1716 & 0.4116 & 0.3291 & 0.0877\\ 0 & 0.6745 & 1.3490 & 2.0235 \end{array}\right].\left(\frac{2.5}{s+2.5}\right)$$ (25)

On the other hand, the PPC was used for the feedback position control as it changes the location of the poles of the uncontrolled system to a stable region. Thus, for precise positioning of the robot, the POS and the T_s_ is set at 1% and 5 s, respectively. Thus, after performing the required computations, the desired poles (*p*) were found as in Eq. 26. Moreover, the control gains, K can be calculated in Matlab using the “place” command, whereby K = place (A,B,p), places the closed-loop poles *p* by calculating a state-feedback control gain matrix K. It can be seen that the poles were placed on left s-place ensuring the stability of the target closed system. Also, this approach considers all the inputs of the system to be control inputs. The algorithm uses the extra degrees of freedom to find an optimal solution that optimizes the sensitivity of the closed-loop poles to changes in the system parameters. Thus, the control gains were calculated as shown in Eq. 27:

p=[-0.8000 ± 0.5458i, -8.0000 ± 5.4575i] (26)

$$K=\left[\begin{array}{cccc} k_{1} & k_{2} & k_{3} & k_{4} \end{array}\right]=\left[\begin{array}{cccc} 0.7440 & -8.2612 & -4.9764 & -0.8322 \end{array}\right]$$ (27)

**FIGURE 6:**
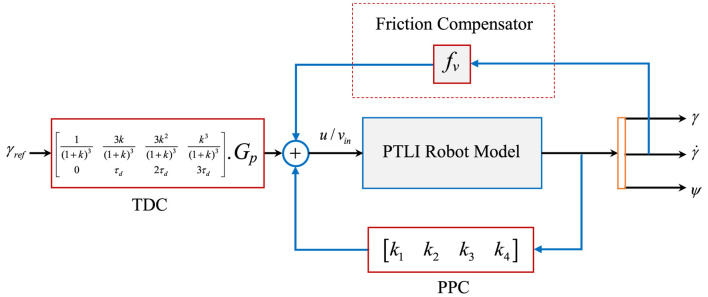
The block representation of the Simulink model of the iTDC + PPC approach.

Once all the required control parameters are found, the iTDC is installed at the input side to shape the signal and the PPC in the feedback path as presented in Eq. 28. The overall block diagram for the simulation analysis is shown in Figure 6.

$$\begin{array}{l} u\left(t\right)=r\left(t\right).TDC.G_{p}-PPC.z\left(t\right)\\ \Rightarrow \left(r\left(t\right)\left[\begin{array}{cccc} 0.1716 & 0.4116 & 0.3291 & 0.0877\\ 0 & 0.6745 & 1.3490 & 2.0235 \end{array}\right].\left(\frac{2.5}{s+2.5}\right)\right)-\left(\left[\begin{array}{cccc} 0.7440 & -8.2612 & -4.9764 & -0.8322 \end{array}\right]\left[\begin{array}{c} \gamma \\ \dot{\gamma }\\ \psi \\ \dot{\psi } \end{array}\right]\right) \end{array}$$ (28)

As shown, the friction compensator is added to counter the effect of the frictional force between the robot and the power line. One of the friction compensation techniques is to add a gain of equal magnitude to the friction components of Eq. 5. This leads to the cancellation of the friction effect. However, the compensator would be removed for the robustness analysis of the controllers to changes of friction coefficients. In sum, the TDC + PPC utilized the advantages of the TDC and PPC for the precise motion control of the sliding robot. The main advantage of the iTDC + PPC approach is that the desired performance of the control system for precise position and oscillation control can be achieved with a little delay penalty. This is possible since the control objectives of the sliding robot, namely, stability, and settling time (braking), can be pre-defined based on the system behaviour.

## Results and Discussion

**FIGURE 7:**
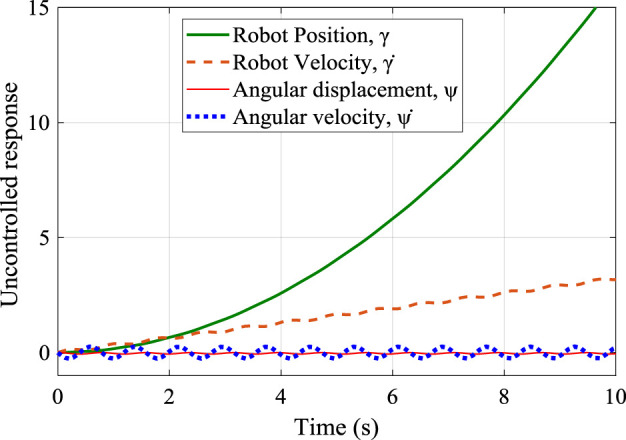
A step response of the uncontrolled system showing its unstable behavior.

In this section, the designed controllers were analyzed using the dynamic equations of Eq. 5 and Eq. 6 in Simulink environment. The response to a step input signal is shown in Figure 7. The position and the speed of the robot are unstable with infinite magnitude, while the angular displacement (sway angle) and its velocity are marginally stable, which reaffirmed the need for effective control.

### Performance Comparison of TDC, PPC, and iTDC + PPC

**FIGURE 8:**
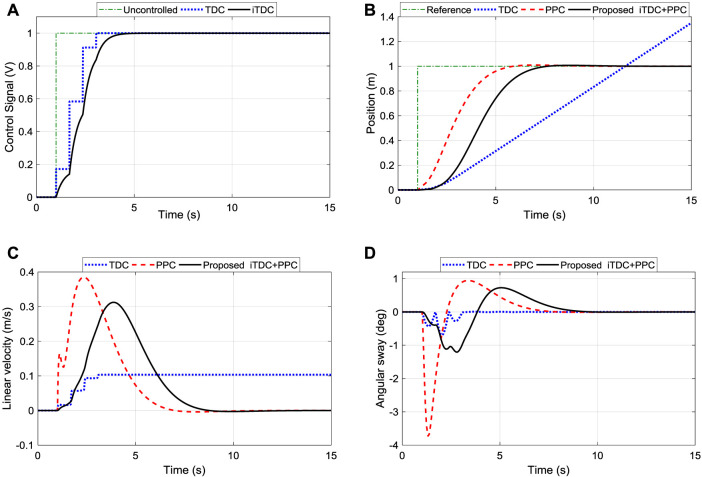
Performance comparison of TDC, iTDC, and iTDC + PPC. **(A)** Control signal of TDC and iTDC showing the improved smooth signal of iTDC; **(B)** Position tracking of the robot showing the unstable response of TDC; **(C)** Linear velocity; **(D)** Sway angle of the arm showing the weak sway control of PPC.

For the performance comparison of the designed controllers, the PPC and the TDC were applied to the system using the Simulink model of Figure 6. Initially, the PPC structure was only implemented without the TDC and then later combined together. As shown, the time delays were added to the feed-forward configuration while the design control gains of the PPC were used for the feedback scheme. Figure 8A shows the control input signal for the convectional TDC and the improved TDC (iTDC). As shown, the iTDC has smoothed the chattering problem associated with the delay constraints of the TDC. Thus, after applying the control signal to the system, the responses of the convectional TDC, iTDC, PPC, and the hybrid iTDC + PPC algorithms were recorded and analyzed. The TDC has poor position control with infinite position response while the PPC and iTDC + PPC remarkably demonstrated a good position and sway control, as shown in Figure 8B. The PPC and iTDC + PPC controllers precisely reached the desired position with no overshoot and settled at about 5 and 6 s, respectively. Note that one second is deducted from the final the settling time since the initial time of applying the control signal was at one second, as shown in the time axis.

Also, the maximum velocity of the TDC (0.1 m/s) was the slowest among the controllers and was maintained infinitely (against the required specifications), as shown in Figure 8C. However, the PPC and iTDC + PPC demonstrated good velocity control and precisely stopped after reaching the desired position. Off course, the effect of the time delay can be seen where the maximum velocity of the PPC (0.3850 m/s) is 271% faster than the velocity of TDC (0.1036 m/s). Nonetheless, the impact of adding the TDC can be observed in Figure 8D, where the maximum sway angle of PPC (−3.7327°) is at least 400% higher than the sway of TDC (−0.6935°). Finally, with the maximum velocity of 0.3125 m/s and sway angle of −1.2066°, the iTDC + PPC has improved the velocity of TDC by 201% and minimizes the angular oscillation of PPC by 209%. Thus, results demonstrated that the proposed iTDC + PPC have utilized the advantages of PPC for position control and the oscillation control of the TDC to achieved precise motion control of the robot.

### Robustness Analysis

In the previous section, it has been established that the iTDC + PPC algorithm improves the control performance of the system. However, the analysis was conducted for the exact parameters of the system. Thus, to further investigates the robustness or sensitivity of the design controllers to variations of system parameters or model uncertainties, the effect of the main parameters of the robot, namely, the length of the suspended arm, the mass of the payload, and the friction coefficient of different surfaces, were analyzed. Here, the trunk (payload) mass, the length of the cylindrical arm, and the friction coefficient were decreased by 50% and then increased by 100%. The analysis is performed within the control system and does not require a separate algorithm. The idea of the analysis is that some system parameters are varied to investigate how the performance of the control system can be affected. Initially, the controllers were designed based on constant values of the system parameters, namely, length, the mass, and the friction coefficient. These parameters are very crucial to the operation of the robot on the power line. Therefore, only the values of the robot parameters were changed while maintaining the same control parameters in the simulation environment. Hence, if the control system is not good enough, changing the values of the parameters will affect the performance of the controllers.

#### Influence of Changing Payload Mass

**FIGURE 9:**
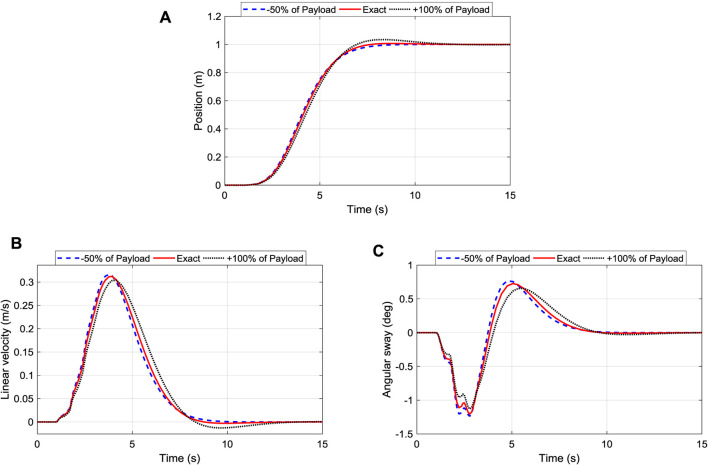
Robustness of iTDC + PPC scheme to changes of payload mass: **(A)** Position Tracking; **(B)** Linear Velocity of the roller; **(C)** Oscillation angle control of the arm.

The operation of the robot could be affected by the mass of the payload as sometimes the onboard electronics component can be added or removed from the trunk. Thus, it is crucial to assess the robustness of the controller to those changes. Therefore, the influence of changing the mass of the payload for the position, velocity, and sway angle of the iTDC + PPC were recorded and analyzed. Figure 9A shows the influence of changing the mass of the payload for the position control of the robot. The system maintained good tracking performance of the desired position. Although, the settling time has been slightly increased from 5.8 to 7.4 s with an overshoot of 4% when the mass of the payload is doubled.

Figure 9B shows the corresponding velocity of the robot, where it is shown that the velocity of the system is maintained within 0.3125 ± 0.008 m/s. Finally, the angular displacement has been maintained as well as shown in Figure 9C, with the maximum sway angle of 1.2066 ± 0.07°. In sum, the responses show that increasing the payload mass slightly increases the oscillation and the overshoot of the system while reducing the robot speed. Nonetheless, the control is robust enough to maintain the variation of payload mass within an acceptable range.

#### Influence of Changing Friction Coefficient

As the robot moves along the power line, which is not smooth, the effect of frictional force cannot be neglected, especially under certain conditions (e.g., dust, snow). In the previous analysis, a friction compensator was added to the system that has a negative magnitude of the frictional force of Eq. 5. However, in this section, the effect of friction is analyzed. Initially, we used a weighing scale to move the robot along the line manually and then record the force required to overcome the static friction, which gives the resulting friction coefficient of 0.155. Then, the coefficient was decreased by 50% and later increased by 100% representing different contact surfaces. Finally, the effects of these three different coefficients for the position, velocity, and sway angle of the TDC, PPC, and iTDC + PPC were analyzed.

**FIGURE 10:**
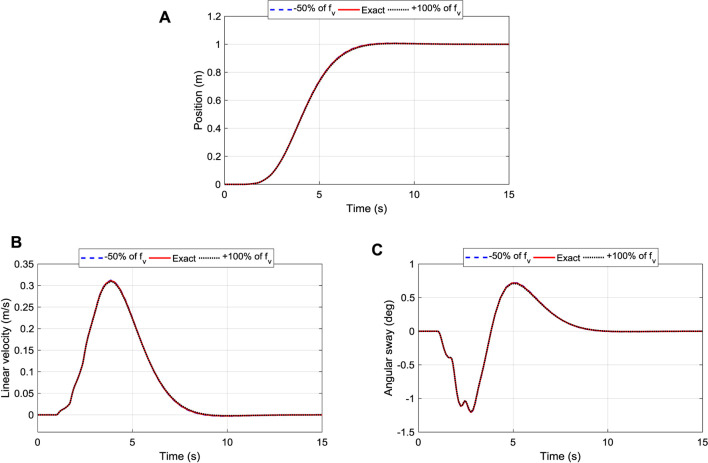
Robustness of iTDC + PPC scheme to changes of friction coefficient: **(A)** Position Tracking; **(B)** Linear Velocity of the roller; **(C)** Oscillation/sway angle control of the arm.

Figure 10A shows the influence of changing the friction coefficient for the position control of the robot. The system maintained good tracking performance of the desired position. The settling time for all the cases have been robustly maintained at 7 s, as shown. The corresponding velocity of the robot shown in Figure 10B illustrates that the velocity of the system is maintained within 0.3109 ± 0.002 m/s. Finally, the angular displacement has been maintained as well as shown in Figure 10C, with the maximum sway angle of 1.2053 ± 0.009°, which shows little effect of the friction force to the controllers.

#### Influence of Changing Arm’s Length

**FIGURE 11:**
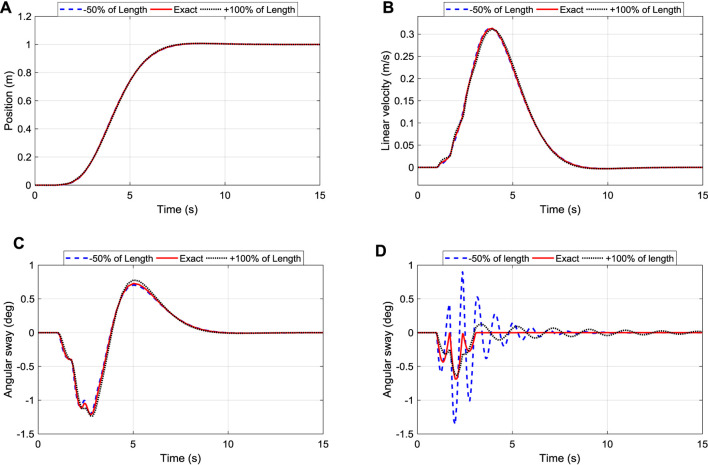
Robustness of iTDC+PPC and TDC to changes of arm length: **(A)** Position Tracking; **(B)** Linear Velocity of the roller; **(C)** Oscillation control of the arm; **(D)** Oscillation control of TDC.

The length of the suspended arm plays an essential role in the automation of the robot. Thus, it is essential to assess its influence on the performance of the designed controllers. The responses of changing the length of the cylindrical arm of the robot (*l*) for the position, velocity, and sway angle of the TDC, PPC, and iTDC + PPC were respectively recorded and analyzed. As shown in Figure 11A, the system maintained good tracking performance of the desired position with a settling time of 6.4 s for the three cases. Figure 11B shows the corresponding velocity of the robot, where it is shown that the velocity of the system is maintained within 0.3850 ± 0.001 m/s. Finally, the angular displacement has been maintained as well as shown in Figure 11C, with the maximum sway angle of 1.2066 ± 0.02°. Thus, it can be seen that the control system is highly robust and insensitive to the changes in arm length.

However, the effect of changing the arm length can be seen for the traditional TDC in Figure 11D, where the angular sway changes drastically unlike the proposed iTDC + PPC. Thus, it is essential to assess the robustness of the traditional controllers. The comprehensive robustness analysis of the three controllers was conducted. The performance criteria, namely, settling time, maximum linear velocity, and maximum angular displacement, were analyzed for different scenarios as summarized in Table 2. As shown, apart from the changes of friction coefficient which has little effect to all the controllers, the changes in payload mass or arm length affects the conventional controllers. Furthermore, r.m.s value which summarized performance of the three controllers were analysed for the angular displacement of the robot. The analysis assess the oscillation or vibration generated for each controller using the three different model parameters.

**TABLE 2:** Robustness performance comparison for TDC, PPC, and iTDC + PPC.

Parameter	Cases	Max. velocity [m/s]			Max. angle [−deg]			R.M.S [deg]		
		TDC	PPC	iTDC + PPC	TDC	PPC	iTDC + PPC	TDC	PPC	iTDC + PPC
Payload (m_t_)	−50%	0.1036	0.4050	0.3166	0.6899	4.1109	1.2433	0.1281	0.8796	0.4873
	Exact	0.1036	0.3850	0.3125	0.6935	3.7327	1.2066	0.1286	0.8131	0.4645
	+100%	0.1036	0.3569	0.3046	0.6931	3.1077	1.1330	0.1283	0.6911	0.4429
Length (*l*)	−50%	0.1062	0.3851	0.3134	1.3711	3.7714	1.2083	0.2865	0.7970	0.4505
	Exact	0.1036	0.3850	0.3125	0.6935	3.7327	1.2066	0.1286	0.8131	0.4645
	+100%	0.1042	0.3838	0.3105	0.6330	3.4892	1.2317	0.1549	0.8639	0.4910
Friction Coef. (f_v_)	−50%	0.1035	0.3841	0.3116	0.6971	3.7280	1.2076	0.1286	0.7984	0.4668
	Exact	0.1034	0.3832	0.3109	0.6966	3.7234	1.2053	0.1288	0.7987	0.4667
	+100%	0.1033	0.3813	0.3092	0.6960	3.7141	1.1959	0.1280	0.8072	0.4538

**FIGURE 12:**
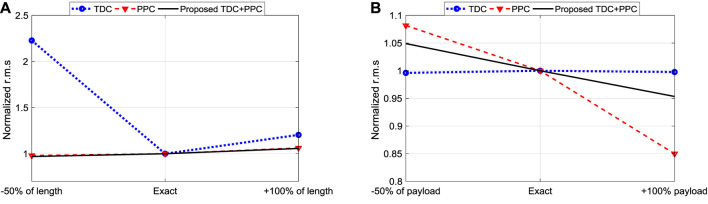
Normalized r.m.s based robustness analysis of TDC, PPC, and iTDC+PPC: **(A)** Change of arm length; **(B)** Change of payload mass.

To clearly compare the robustness of the controllers, the r.m.s values were normalized about the results of the exact parameters for the three scenarios. Here, the deviation from unity show how sensitive a particular control is to parameter variations. Note that the TDC cannot provide position control. Figure 12 show the visualization of the robustness analysis of the three controllers. The PPC is insensitive to the change in length while the TDC is highly affected. In contrast, the TDC shows robustness to the change of payload while the performance of PPC is weak. Thus, our notion of improving the TDC and hybridizing it with the PPC showed a promising robustness for effective automation of the power line inspection robot.

## Conclusion

The main objective of this work is to investigate an approach for precise motion control of a dual-arm inspection robot based on the possible application of two controllers, namely the improved input shaping time delay control (iTDC) and the state feedback-based pole placement control (PPC). The controllers were designed and implemented on the system in Matlab software. Then, the robustness analysis to the changes of three parameters, namely, the length of the suspended arm, the mass of the payload, and the friction coefficient of different surfaces, were analyzed. Finally, the simulation responses of the control system were recorded and analyzed based on the final settling time, maximum linear velocity, and angular oscillation of the robot. The conclusion of the complete study is summarized as follows:

1) The dynamic model of the inspection robot on the power line was derived using the Lagrangian equations, and the controllers were implemented on the model.
2) Although the PPC has demonstrated good position tracking, adding the iTDC significantly improves the sway angle suppression with little delay penalty.
3) The iTDC + PPC has improved the velocity of TDC by 201% and minimizes the angular oscillation of PPC by 209%. Thus, results demonstrated that the proposed iTDC + PPC have utilized the advantages of PPC for position control and the oscillation control of the TDC to achieved precise motion control of the robot
4) The robustness analysis showed that changes of the arm’s length had the smallest influence on the controller, followed by friction and mass of the payload. In each case, the controller showed strong robustness to parameter variations.

Finally, the simulation study of the selected control algorithms demonstrated a guaranteed stability and robustness for the precise motion control of the inspection robot. Our future work will focus on implementing the controller on the lab-scale dual-arm robot for real-time applications.

## Data Availability

The raw data supporting the conclusions of this article will be made available by the authors, without undue reservation.
